# Mutation Screening of 1,237 Cancer Genes across Six Model Cell Lines of Basal-Like Breast Cancer

**DOI:** 10.1371/journal.pone.0144528

**Published:** 2015-12-15

**Authors:** Eleonor Olsson, Christof Winter, Anthony George, Yilun Chen, Therese Törngren, Pär-Ola Bendahl, Åke Borg, Sofia K. Gruvberger-Saal, Lao H. Saal

**Affiliations:** 1 Division of Oncology and Pathology, Department of Clinical Sciences, Lund University, Lund, Sweden; 2 Lund University Cancer Center, Lund, Sweden; 3 CREATE Health Strategic Centre for Translational Cancer Research, Lund University, Lund, Sweden; CNR, ITALY

## Abstract

Basal-like breast cancer is an aggressive subtype generally characterized as poor prognosis and lacking the expression of the three most important clinical biomarkers, estrogen receptor, progesterone receptor, and HER2. Cell lines serve as useful model systems to study cancer biology *in vitro* and *in vivo*. We performed mutational profiling of six basal-like breast cancer cell lines (HCC38, HCC1143, HCC1187, HCC1395, HCC1954, and HCC1937) and their matched normal lymphocyte DNA using targeted capture and next-generation sequencing of 1,237 cancer-associated genes, including all exons, UTRs and upstream flanking regions. In total, 658 somatic variants were identified, of which 378 were non-silent (average 63 per cell line, range 37–146) and 315 were novel (not present in the Catalogue of Somatic Mutations in Cancer database; COSMIC). 125 novel mutations were confirmed by Sanger sequencing (59 exonic, 48 3’UTR and 10 5’UTR, 1 splicing), with a validation rate of 94% of high confidence variants. Of 36 mutations previously reported for these cell lines but not detected in our exome data, 36% could not be detected by Sanger sequencing. The base replacements C/G>A/T, C/G>G/C, C/G>T/A and A/T>G/C were significantly more frequent in the coding regions compared to the non-coding regions (OR 3.2, 95% CI 2.0–5.3, P<0.0001; OR 4.3, 95% CI 2.9–6.6, P<0.0001; OR 2.4, 95% CI 1.8–3.1, P<0.0001; OR 1.8, 95% CI 1.2–2.7, P = 0.024, respectively). The single nucleotide variants within the context of T[C]T/A[G]A and T[C]A/T[G]A were more frequent in the coding than in the non-coding regions (OR 3.7, 95% CI 2.2–6.1, P<0.0001; OR 3.8, 95% CI 2.0–7.2, P = 0.001, respectively). Copy number estimations were derived from the targeted regions and correlated well to Affymetrix SNP array copy number data (Pearson correlation 0.82 to 0.96 for all compared cell lines; P<0.0001). These mutation calls across 1,237 cancer-associated genes and identification of novel variants will aid in the design and interpretation of biological experiments using these six basal-like breast cancer cell lines.

## Introduction

Among women, breast cancer is the most common malignancy and a leading cause of death with nearly 1.7 million cases diagnosed worldwide and over 500 thousand deaths every year [1]. A minority of these cases, 5–10%, are caused by mutations in high-penetrance germline loss-of function genes (*BRCA1*, *BRCA2*) or in low-penetrance susceptibility genes/regions, although not all of the hereditary genetic factors have been identified [2–5]. However, the vast majority of breast cancers result from mutations acquired by aging and lifestyle and environmental factors acting in combination with genetic predisposition [6, 7].

Molecular characterization has shown that breast cancer is a highly heterogeneous disease that can be divided into at least four well-defined subtypes: the hormone receptor positive subtypes, luminal A and luminal B, the HER2 subtype enriched for cases with HER2 amplification, and the basal-like subtype which usually lacks expression of the estrogen (ER), progesterone (PR), and HER2 receptors (so called “triple-negative”) [8–10]. Basal-like breast cancers, which comprise 10–20% of all breast cancers, are also typically high grade, highly proliferative, genomically unstable, and have frequent somatic mutation of *TP53* as well as loss of PTEN expression, and express basal cytokeratins such as CK5 and CK14 [8, 10–12]. Interestingly, hereditary *BRCA1*-mutations appears to primarily predispose for the development of basal-like breast cancers [13], indicating that BRCA1 dysfunction is a potent driver of basal-like tumorigenesis. Since basal-like tumors rarely express ER/PR/HER2, there is no recommended specific targeted therapy in current clinical practice, and generally these tumors exhibit poor prognosis features as described above. Therefore, there is a great demand for an improved understanding of basal-like breast cancer biology and for the development of drug targets for this aggressive subtype.

Recently, massively parallel sequencing has opened up possibilities to perform integrated large-scale screening studies ranging from the detection of somatic structural variants to the cataloguing of variants at single base resolution. These studies have given us better understanding of which molecular mechanisms and signaling pathways are aberrant in many types of solid tumors, and also to some extent within different subtypes of breast cancer [14–16]. Nonetheless, the number of recurrently mutated genes in the basal-like breast cancer subtype has been sparse but notable for a high frequency of *TP53* mutations (84%); the next most commonly mutated gene is *PIK3CA* (7%) [14]. The use of model systems *in vitro* and *in vivo* will be integral to the identification of new drug targets and development of improved therapeutic options for basal-like breast cancer. To draw reliable conclusions from these models under any experimental condition, it will be important to be cognizant of the genomic context of each model.

In this study, our aim was to improve the characterization of the mutational pattern of basal-like breast cancer model cell lines. A custom panel of 1,237 cancer-associated genes was used to perform hybrid capture and deep sequencing on six commonly utilized publically-available basal-like cancer cell lines (HCC38, HCC1143, HCC1187, HCC1395, HCC1954, and HCC1937) with matched normal DNA to identify both known and novel gene mutations. Moreover, we evaluated the use of targeted resequencing data to estimate the DNA copy number profile in the regions of interest.

## Results and Discussion

### Detection of somatic variants in breast cancer cell lines

To characterize their mutational profiles, the breast cancer cell lines HCC38, HCC1143, HCC1187, HCC1395, HCC1937, HCC1954 (previously classified as basal-like [17, 18]), as well as their matched normal lymphocyte cell lines, were analyzed using targeted DNA capture and massively-parallel single-read DNA sequencing. To maintain the mutational profile of the originating cell line population, all cell lines were obtained directly from the American Type Culture Collection repository and analyzed at very low passage. A custom SureSelect library (Agilent Technology) targeting all coding exons for 1,237 cancer-associated genes, a portion of each gene’s 5’UTR and upstream region, and the entire 3’UTR, was designed and used to capture approximately 6.5 Mbases of the human genome (see Methods) (S1 Table). For the tumor and normal cell line DNAs, targeted regions were captured and PCR-enriched and the libraries sequenced on an Illumina Genome Analyzer IIx instrument. For the cancer cell lines and matched normal samples, on average 6.4 million (range 5.9–7.1) and 5.7 million (range 3.7–6.9) unique 75–80 bp reads were sequenced, respectively. The mean coverage of the targeted area was 127-fold (range 96–152) for the cancer samples and 98-fold (range 87–127) for the normal samples (S2 Table). Across all samples, on average 90% (range 88–92%) of the targeted regions had a sequence coverage of 15 reads or more (S2 Table).

The Burrows-Wheeler aligner (bwa) [19] was used for the alignment of sequencing reads to the reference human genome (hg19), and Genome Analysis ToolKit (GATK) UnifiedGenotyper was used for the calling of single nucleotide variants (SNVs) and indels [20]. A mean of 5,494 SNVs (range 5,302–5,740) and 643 indels (range 572–685) that passed the variant calling filter were identified for each tumor-normal pair (Table 1), and in total 36,822 variants were called of which 95.7% were known single nucleotide polymorphisms present in dbSNP138. To check the robustness of our method we compared our variant calls to previously reported mutations for these cell lines that had been curated in the COSMIC database version 68 [21, 22]. Of 479 somatic mutations in COSMIC in genes that we targeted, 412 (86.0%) were identified in our data as perfect matches (S3 Table). When allowing for loosely matching variants to COSMIC (*i*.*e*., the position was shifted a few bases or alternatively the GATK UnifiedGenotyper detected a substitution as two separate events), an additional 12 variants could be matched increasing the detection rate to 88.5% (424 of 479). Of note, we detected 94.3% of all SNVs (412 of 437) whereas only 28.6% (12 of 42) of indels were identified. These results are in line with single read data being suboptimal for detection of longer indels, and the bwa aligner and UnifiedGenotyper variant caller GATK pipeline being less than ideally suited for indel calling in tumor genomes [23]. The majority of COSMIC matching variants (83.3%) were qualified as “high confidence” somatic variants (see Methods).

**Table 1:** All called SNVs and indels.

Cancer cell line	HCC1143	HCC1187	HCC1395	HCC1937	HCC1954	HCC38	Average	Total
Matched normal cell line	HCC1143BL	HCC1187BL	HCC1395BL	HCC1937BL	HCC1954BL	HCC38BL		
# of SNPs called (passing variant call filter in GATK)	5302	5426	5740	5517	5557	5421	5494	32963
# of Indels called (passing variant call filter in GATK)	572	673	685	621	664	644	643	3859
# of SNPs and indels (passing variant call filter in GATK)	5874	6099	6425	6138	6221	6065	6137	36822
# of somatic heterozygous variants ("high confidence" variants in GATK)	51	49	198	87	54	51	82	490
# of somatic homozygous variants ("high confidence" variants in GATK)	10	23	88	31	4	12	28	168
In dbSNP138 (SNPs >1% minor allele frequency (MAF), non-clinically associated, passing variant call filter in GATK)	5670	5845	6061	5912	5904	5830	5870	35222
Total # of somatic variants ("high confidence" variants in GATK)*	61 (21)	72 (18)	286 (155)	118 (77)	58 (19)	63 (25)	110	658 (315)
Exonic*	46 (6)	62 (8)	170 (49)	69 (31)	41 (3)	49 (13)	73	437 (110)
frameshift deletion*	1 (1)	1 (0)	1 (1)	0	1 (0)	2 (1)	1	6 (3)
frameshift insertion*	0	4 (2)	0	0	1 (0)	0	1	5 (2)
nonframeshift deletion*	0	2 (0)	0	0	0	0	0	2 (0)
nonsynonymous SNV*	37 (2)	46 (3)	133 (26)	46 (16)	33 (0)	38 (9)	56	333 (56)
stopgain SNV*	3 (0)	5 (0)	6 (0)	5 (1)	1 (0)	1 (0)	4	21 (1)
stoploss SNV*	0	0	0	0	0	1 (0)	0	1 (0)
synonymous SNV*	5 (3)	4 (3)	29 (21)	18 (14)	5 (3)	7 (3)	11	68 (47)
Unknown*	0	0	1 (1)	0	0	0	0	1 (1)
Splicing*	0	0	6 (0)	2 (1)	1 (0)	1 (0)	2	10 (1)
UTR3*	5 (5)	4 (4)	26 (26)	18 (18)	3 (3)	3 (3)	10	59 (59)
UTR5*	1 (1)	0 (0)	8 (8)	4 (4)	2 (2)	2 (2)	3	17 (17)
Others (intronic, upstream, downstream, intergenic, ncRNA)*	9 (9)	6 (5)	76 (72)	25 (23)	11 (11)	8 (7)	23	135 (128)
# of somatic variants, non-silent ("high confidence" variant calls in GATK)*	41 (3)	58 (5)	146 (27)	53 (18)	37 (0)	43 (10)	63	378 (63)

*Novel variants in parenthesis

In total, 658 high confidence somatic variants were identified across the six basal-like cell lines, and of these, 490 were heterozygous, 168 were homozygous, and 378 were non-silent (count of non-silent: HCC1143 n = 41, HCC1187 n = 58, HCC1395 n = 146, HCC1937 n = 53, HCC1954 n = 37, HCC38 n = 43) (Table 1). The non-silent variants were defined as exonic indels, non-synonymous SNVs, stopgain or stoploss SNVs and splicing mutations. Out of the 658, 315 variants were novel mutations (not present in COSMIC) and of these 110 were exonic and 63 were non-silent. Novel non-coding mutations were also found in the 3’UTR (n = 59), 5’UTR (n = 17), and within 1000 bp upstream of the gene transcriptional start site (n = 20) (Table 1 and S3 Table). Conversely, of the 343 high confidence somatic variants present in COSMIC (S3 Table), 315 were non-silent.

**Fig 1:**
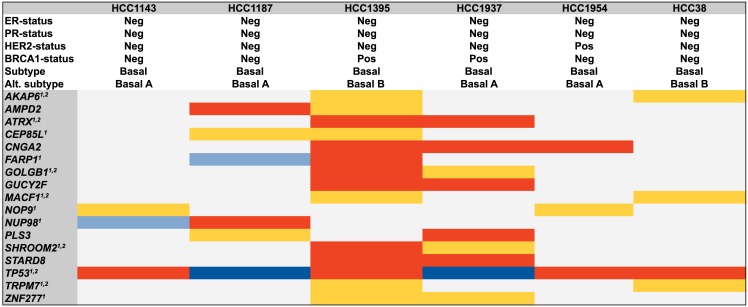
Recurrently mutated genes in 6 basal-like cell lines. The 17 genes with identified somatic mutations in two or more cancer cell lines are shown, colored according to mutation type. Heterozygous non-synonymous single nucleotide variants (SNV) are in yellow; homozygous non-synonymous SNVs are in red; heterozygous stopgain/short indels mutations are in light blue; homozygous stopgain/short indels mutations are in blue. ^1^Non-synonymous SNVs, stopgain SNVs or short indels also identified in a breast cancer study from TCGA (basal-like tumors) [14]. ^2^Non-synonymous SNVs, stopgain SNVs or short indels also identified in a breast cancer study of triple-negative tumors [28]. The subtype classification and receptor status have been described earlier [17, 18, 24].

In line with the high mutation rate of *TP53* in basal-like breast cancer [14], *TP53* was mutated in all six basal-like cell lines analyzed [17, 24–26], and in total, there were 17 genes with non-synonymous somatic high confidence mutations in two or more of the tumor cell lines (Fig 1). For example, three different homozygous mutations were detected in the *CNGA2* gene: two had not been previously described for the *BRCA1*-deficient cell lines HCC1395 and HCC1937, and the third was a known homozygous mutation (in COSMIC) in HCC1954. Additionally, two of the cell lines had non-coding *CNGA2* variants: in HCC1143 there was also a somatic mutation upstream, and HCC1395 was mutated in the 3’UTR.In both of the *BRCA1*-deficient cell lines a novel non-synonymous homozygous mutation in *GUCY2F* (Xq22, exon3:c.T851C:p.L284P) was found, and additionally the genes *ATRX* (Xq21.1), and *STARD8* (Xq13.1), *GOLGB1* (3q13), *SHROOM2* (Xp22.3), and *ZNF277* (7q31.1) all harbored non-synonymous mutations. In the cell lines HCC38 and HCC1395, classified as basal B in the work by Neve et al [17], the *AKAP6* (14q12), *MACF1* (1p32-p31) and *TRPM7* (15q21) genes all had non-silent mutations (Fig 1). For these genes, it was recently reported that among 81 basal-like breast tumors, 5% had somatic *GOLGB1* mutations and 4% harbored mutations in *AKAP6* or *MACF1* [14, 27]. In another study of triple-negative tumors [28], *AKAP6*, *MACF1*, and *TRPM7* were mutated in 3% out of 65 analyzed tumors. In the same cohort, *MYO3A* was mutated in 9% of the cases and we also found two non-silent mutations in that gene in the HCC38 cell line.

### Sanger sequencing validation

Selecting for exonic, 3’UTR, and 5’UTR variants, 187 SNVs and short indels from the exome sequencing results were validated by Sanger sequencing of the cell line tumor-normal pairs (S4 and S5 Tables). Out of the 187, 130 were high confidence and 57 were low confidence somatic variants with quality-by-depth (QD) values >3. The low confidence variants were Sanger sequenced to examine the utility of sub-optimal variant calls (S4 and S5 Tables). Of 187 mutations examined, 172 were not present in COSMIC (including 85 exonic, 19 5’UTR, 60 3’UTR, 1 splice site, and 7 intronic variants; S4 Table) and 15 were present in COSMIC (with 6 of these variants found by GATK UnifiedGenotyper at slightly different positions in our data; S5 Table).

Of the 172 novel candidates (not in COSMIC), 118 were high confidence variants. Of these, 111 (94.1%) were validated as somatic, 5 (4.2%) were wildtype, none were germline, and 2 validated with a different mutation: one SNV variant was homozygous instead of heterozygous (at the same position) and one SNV was a 15 bp deletion in *PLCD3* with Sanger sequencing (also present in COSMIC, COSM50189). In total, 98% of the exonic, 93.9% of the 3’UTR, and 81.8% of the 5’UTR variants could be validated as somatic (Table 2 and S4 Table). However, only 25.9% (14 of 54) of the low confidence novel candidates could be validated as somatic: 29.6% were germline and 44.4% were determined as wildtype (Table 2 and S4 Table). Thus, we found that the majority of high confidence variants could be verified, whereas there are few true positives among the lower confidence somatic variant calls.

**Table 2:** Sanger sequencing validation of novel SNVs and indels.

Cancer cell line	HCC1143	HCC1187	HCC1395	HCC1937	HCC1954	HCC38	Total	Validation rate (%)
Matched normal cell line	HCC1143BL	HCC1187BL	HCC1395BL	HCC1937BL	HCC1954BL	HCC38BL		
# of novel somatic variants validated vs. analyzed by Sanger (good quality, loose), "high confidence" variant calls in GATK	7/8	7/7	49/54	40/40	1/2	7/7	111/118	94.1
exonic	3	4	21	20	0	2	50	98.0
frameshift deletion	1		1				2	100
frameshift insertion		2					2	100
nonsynonymous SNV	2	2	20	13		2	39	97.5
stopgain SNV				1			1	100
synonymous SNV				6			6	100
splicing				1			1	100
UTR3	4	3	19	16	1	3	46	93.9
UTR5			5	3		1	9	81.8
Others (Intronic, ncRNA, unknown)	0	0	4	0	0	1	5	83.3
# of novel somatic variants validated vs. analyzed by Sanger (good quality, loose), "low confidence" variant calls in GATK	3/13	0/6	6/14	2/7	1/4	2/10	14/54	25.9
exonic	1		4	2	1	1	9	27.3
nonsynonymous SNV	1		3	2	1	1	8	25.8
stopgain SNV			1				1	50
UTR3	1		1				2	18.2
UTR5						1	1	12.5
Others (Intronic, ncRNA, unknown)	1		1				2	100
Total # of novel somatic variants validated by Sanger (good quality, loose), "low confidence"+ "high confidence" calls in GATK	10	7	55	42	2	9	125	72.7

Across the six cell lines, 125 novel somatic variants (111 high confidence and 14 low confidence) were confirmed by Sanger sequencing and of these 59 were exonic, 48 were in 3’UTRs, 10 in 5’UTRs, 1 affected splicing, and 7 mutations were located in ncRNA or intronic regions. Of the novel somatic mutations within exons, 47 were non-synonymous, 6 were synonymous, 2 were stopgain, and 4 were frameshift indels (Table 2 and S4 Table). Of the 15 selected variants identified by our pipeline but also present in COSMIC, 12 were high confidence variants whereof the Sanger sequencing results verified all 12: 8 exactly and 4 matched loosely to the COSMIC positions (<5bp) (due to location in a homopolymer region). Two of the three remaining low confidence variants were determined by Sanger sequencing to be a single 2-bp deletion (matching the COSMIC variant COSM20994), and the third was confirmed by Sanger (matched to both COSMIC and our UnifiedGenotyper call) (S5 Table).

We also selected for Sanger sequencing 36 known variants that were present in COSMIC for these cell lines but not detected by our pipeline (Table 3 and S6 Table). Of these, 10 mutations were determined by Sanger sequencing to be in fact wildtype in our cell lines, 1 variant was germline, and 2 COSMIC variants were found to be a different somatic mutation at the same locus. On the other hand, 23 of these COSMIC variants (63.9%) were confirmed to be present by Sanger sequencing, thus representing false-negative calls by our pipeline (S6 Table). To note, out of the COSMIC variants missed by our targeted sequencing data pipeline but confirmed by Sanger, 52% (12 out of 23) were deletions and 7 of these were >10bp (S6 Table).

**Table 3:** Sanger sequencing validation of SNVs and indels in COSMIC v68, not detected by GATK.

Cancer cell line	HCC1143	HCC1187	HCC1395	HCC1937	HCC1954	HCC38	Total	Validation rate (%)
Matched normal cell line	HCC1143BL	HCC1187BL	HCC1395BL	HCC1937BL	HCC1954BL	HCC38BL		
# of variants in Cosmic v68 not detected by GATK validated vs analyzed by Sanger	2/3	4/5	4/10	6/8	3/5	4/5	23/36	63.9
exonic	2	4	4	6	3	3	22	62.9
frameshift deletion	0	3	1	2	0	1	7	87.5
nonframeshift deletion	0	0	2	0	1	1	4	100
nonsynonymous SNV	2	1	1	4	2	1	11	55.0
intergenic	0	0	0	0	0	1	1	100

Previous mutation screening studies including these six cell lines have focused on detecting mutations only in coding regions, which explain the relatively high numbers of novel mutations outside these regions in our data. However, the fact that we could detect novel mutations in coding regions in cell lines previously analyzed by others could possibly be explained by differences in sequencing technology, on-target efficiency and sequence coverage, as well as analysis methodologies [22, 25, 29, 30].

### Integrated mutational and copy number data

Copy number variation (CNV) across the targeted regions was calculated using the software CONTRA [31]. CONTRA was specifically developed for analysis of copy number variation in resequenced data and is designed to handle GC-content bias and differences in sequencing depth in the input samples. The adjusted mean log ratios derived from CONTRA were segmented with the Bioconductor package GLAD [32] and copy number estimations (log2 ratios) for individual genes were computed for each tumor cell line (S7 Table).

**Fig 2:**
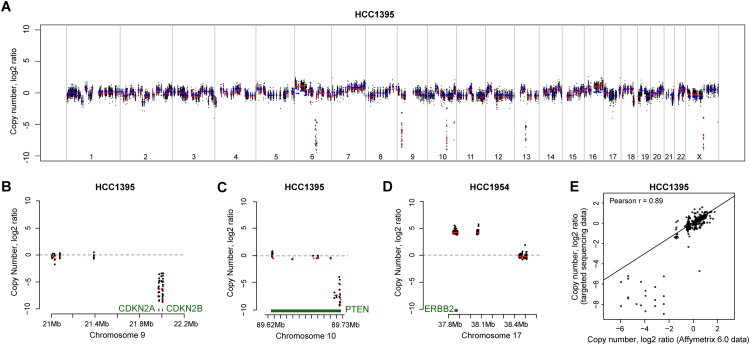
Copy number estimation using targeted sequencing data. (a) Whole genome plot of HCC1395 copy number variations derived from targeted sequencing data analyzed with CONTRA in comparison to segmented Affymetrix 6.0 copy number data for the same cell line. For the sequencing data, the black datapoints are the CONTRA adjusted mean log2 ratios, and red datapoints are the CONTRA/GLAD segmented copy log2 ratios. For the Affymetrix 6.0 data, blue datapoints are the segmented copy number data. Zoomed-in plots of three selected genes, (b) *CDKN2A* and (c) *PTEN* in HCC1395, and (d) *ERBB2* in HCC1954 (color codes are as above). (e) Representative correlation plot for one cell line, HCC1395, of segmented CONTRA copy number data versus Affymetrix 6.0 segmented copy number data (Pearson r = 0.89). Correlation plots for all cell lines are presented in S2 Fig.

To validate our copy number estimates, publicly available segmented copy number data generated using the Affymetrix 6.0 SNP array platform was downloaded for these cell lines [16]. The targeted exome-derived CNV data and Affymetrix CNV data are plotted in Fig 2a and S1 Fig. Zoomed-in plots of three selected genes, *CDKN2A* and *PTEN* (in HCC1395), and *ERBB2* (HCC1954) are shown in Fig 2b–2d. In general, there was a good concordance in copy number estimation between the two methods (Pearson correlations between 0.82 and 0.96 per cell line; P-values<0.0001; Fig 2e and S2 Fig) despite the sparse coverage (0.22%) of the genome in the exome sequencing data.

**Fig 3:**
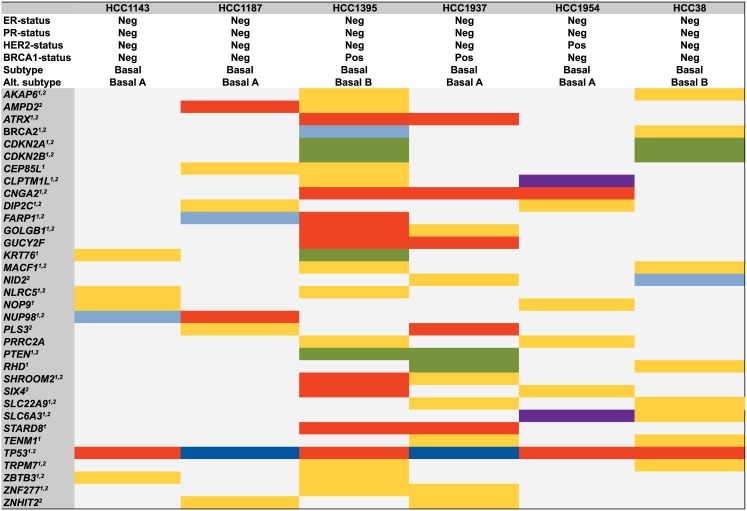
Integrated recurrent somatic mutations and copy number variations. The 34 genes affected by mutation and/or copy number gain or deletion (|log2 ratio|>2) in two or more cell lines are shown. Color codes are as in Fig 1, with the addition that large homozygous deletions are in green and amplifications are in purple. ^1^Non-synonymous SNVs, stopgain SNVs, short indels respective deep deletions and amplifications also identified in a breast cancer study from TCGA (basal-like tumors) [14]. ^2^Non-synonymous SNVs, stopgain SNVs, short indels respective homozygous deletions and amplifications also identified in a breast cancer study of triple negative tumors [28]. The subtype classification and receptor status have been described earlier [17, 18, 24].

Mutational profiles and copy number data were integrated to get a comprehensive summary of the genomic context in the targeted regions of the six breast cancer cell lines. All novel high confidence non-silent somatic mutations, known variants in COSMIC also detected in our analyses, mutations confirmed as somatic with Sanger sequencing, as well as genes with high level amplification or with exonic deletions (i.e., |log2 ratios|>2) were included. In Fig 3, all genes with any of the above somatic genetic aberrations in two or more cancer cell lines are presented. For example, known large deletions in the *PTEN* gene were detected in both of the *BRCA1-*deficient cell lines HCC1395 and HCC1937. Moreover, known large deletions of *CDKN2A* and *CDKN2B* and heterozygous SNVs in the *BRCA2* gene were found in both of the basal B cell lines HCC38 and HCC1395 (the *BRCA2* mutation was novel in HCC38). In the cancer cell lines classified as basal A [17], the gene *NOP9* was mutated in HCC1143 and HCC1954, and *ZNHIT2* was mutated in both HCC1937 and HCC1187. Out of the genes shown in Fig 3, 91% (74 of 81) had genetic alterations in terms of copy number changes or somatic mutations in a study of 81 triple-negative tumors [14] in the genes *BRCA2* (11%), *CDKN2A* (11%), *CDKN2B* (12%), *DIP2C* (12%), *FARP1* (5%), *SLC6A3* (5%), *PTEN* (6%), as well as *TP53* (85%).

The integrated somatic aberrations were annotated according to signaling pathways [33]. The Notch signaling pathway was affected in 4 of 6 cell lines with non-silent mutations present in *NOTCH4* in HCC1395, *DTX3L* and *MAML3* in HCC1937, *NOTCH1* in HCC1954, and also high-level amplifications of *JAG2* and *NOTCH3* in HCC1143. Notch signaling is a complex and highly conserved pathway that may be either tumor suppressive or oncogenic depending on the cellular context, and may be a therapeutic target for basal-like breast cancer [34, 35]. Genes involved in the phosphatidylinositol signaling system that harbored mutations were *PLCG1* in HCC1395, *PLCB2* in HCC1937, *DGKE* and *PIK3CA* in HCC1954, *DGKG* in HCC1395, *PLCB1* in HCC38, and also large homozygous deletions of *PTEN* in HCC1395 and HCC1937. The phosphatidylinositol signaling system is well known to be of importance in breast cancer and regulates key components of proliferation and apoptosis [12]. In the KEGG pathway for regulation of the cytoskeleton, genes that harbored mutations were *FGFR2*, *RAC2*, and *VAV3* in HCC1143; *ARHGEF4*, *ITGB2*, *MYH9*, and *PPP1R12A* in HCC1187; *FGFR1*, *ITGA9*, *PDGFRB*, and *BCAR1* in HCC1395; *CFL2* and *PIK3CA* in HCC1954; and genes with high level amplifications were *FGF3* and *FGF4* in HCC1143, and *FGFR4* in HCC1954. The role of these gene mutations requires further study, however it is intriguing as reorganization of the actin cytoskeleton could affect focal adhesion stability and is also associated with epithelial-to-mesenchymal transition (EMT), a process thought to increase metastatic potential and enriched in basal-like breast cancer [36, 37].

### Predicted effects of somatic mutations

We annotated the high confidence somatic SNVs with three different programs and the complete annotation results are presented in S3 Table. We used Mutation Assessor [38] to categorize the non-synonymous SNVs as “functional” (i.e., deleterious; 35.3%, n = 116) or “non-functional” (64.7% n = 213). To note, Mutation Assessor only scores the non-synonymous SNVs. A substantial part of the non-functional variants (114 of 213) in Mutation Assessor were annotated as “disease causing” by another software, MutationTaster [39]. Non-synonymous, synonymous, and nonsense mutations are scored by this software and 67.4% (250 out of 371) of these were annotated as disease causing. Moreover, 26 (7.0%) SNVs were assigned as “polymorphisms” by MutationTaster even if they were determined as somatic in our data (but actually present in dbSNP). In total, 101 variants were categorized as both functional in Mutation Assessor and disease causing in MutationTaster. Using Combined Annotation Dependent Depletion (CADD) [40], the mutations were scored for deleteriousness along a continuous scale, and stopgain SNVs were exclusively top ranked. The CADD scores were significantly higher for MutationTaster disease causing variants compared to the polymorphisms (P<0.0001, Mann-Whitney U test) and similarly for the Mutation Assessor functional compared to non-functional variants (P<0.0001, Mann-Whitney U test). Importantly, CADD also scores most variants outside exonic regions. Frameshift indels were not classified by any of the three programs (indels cannot be matched to CADD database in Annovar).

### Base replacement patterns in coding versus non-coding regions

The transitions to transversions ratio (T_i_/T_v_) for known SNPs in the targeted regions (including variants not passing variant call filters) was on average 2.66 for all cell lines. Since the targeted regions contain both coding and non-coding regions, this suggests a low false positive variant call rate in our targeted sequencing data [41]. Considering only the high confidence somatic variants, the mutational frequency was on average higher in the coding regions (20.6 mutations/Mbp, range 11.6–48.0) than in the non-coding regions (8.7 mutations/Mbp, range 1.7–19.9) and the *BRCA1*-deficient HCC1395 cell line appeared to exhibit a hypermutated genotype. Interestingly, the average T_i_/T_v_ ratio for somatic variants in the coding regions was lower at 0.94 (range 0.62–3.88) and the *HER2*-positive cell line (HCC1954) had the highest ratio, primarily owing to a high proportion of C/G→T/A transitions. For the non-coding somatic variants, the average T_i_/T_v_ ratio was 1.32 (range 0.57–1.81), and HCC1954 showed the lowest ratio. Possibly, disparity in the selection pressure and in the efficiency of the repair mechanisms could explain the differences in ratios between coding and noncoding regions.

**Fig 4:**
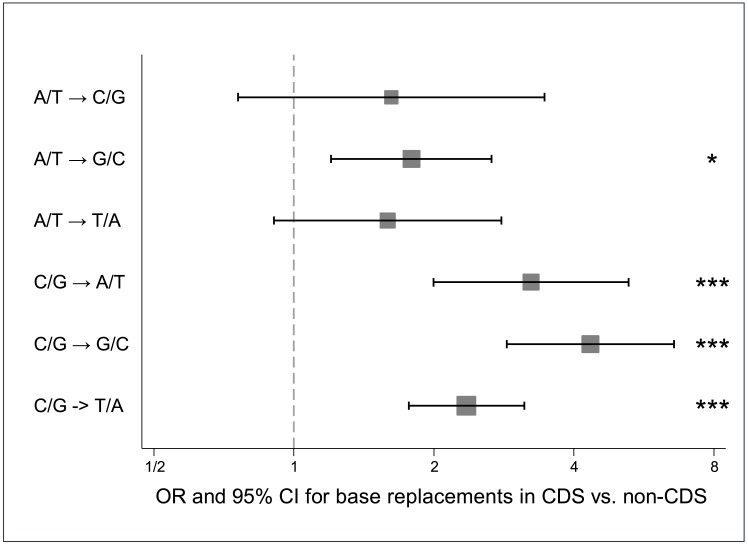
Base replacements in coding regions versus non-coding regions. Forest plot indicating odds ratio (marker) and 95% confidence intervals (whiskers) for the frequency of indicated base replacements in coding regions (CDS) versus non-coding (non-CDS) regions. The dimensions of the squares are inversely proportional to the standard error (SE) of ln(odds ratio). * P = 0.024; *** P<0.0001 (Bonferroni adjusted).

Of the high confidence somatic SNVs, a G/C base pair was replaced in 76.4% and 61.0% of instances within the coding and non-coding regions, respectively. Differences in the GC-content of the targeted regions, 51.1% in the coding and 47.0% in the non-coding regions, do not explain the bias. For the somatic variants, C/G→T/A transitions were the most common in both coding (34.9%) and non-coding regions (35.1%) on average for all cell lines. The second most common base replacement in the coding regions was C/G→G/C transversions (26.2%) whereas A/T→G/C transitions were the next most common in the non-coding regions (21.5%). Accounting for differences in GC-content, the base replacements C/G→A/T, C/G→G/C, C/G→T/A and A/T→G/C were significantly more frequent in the coding regions compared to the non-coding regions (OR 1.8, 95% CI 1.2–2.7, P = 0.024; OR 3.2, 95% CI 2.0–5.3, P<0.0001; OR 4.3, 95% CI 2.9–6.6, P<0.0001; OR 2.4, 95% CI 1.8–3.1, P<0.0001, respectively) (Fig 4, S8 Table).

In a recent study, specific signatures for mutational processes were suggested for breast cancer, and the most common signatures (Signatures 1B, 2 and 3) involved C/G→T/A, C/G→G/C and C/G→A/T replacements with an overall prominence for C/G→T/A transitions (no comparison between coding and non-coding regions were available) [42]. Notably, Signature 3 is characterized by enrichment for C/G substitutions, and one could speculate that this signature is associated with the mutational pattern we detected in the coding regions. Interestingly, Signature 3 has previously been associated with cases harboring *BRCA1* and *BRCA2* mutations [42].

The highest fraction of the C/G→T/A substitutions (59.0% of all SNVs) was detected in the coding regions of the *HER2*-amplified cell line HCC1954 and this pattern partly resembles that of APOBEC mutagenic activity, which is commonly aberrant in many types of human cancers. Of the C/G→G/C substitutions, which have also been suggested to be caused by APOBEC editing, HCC1954 had the lowest fraction (7.7%) and instead the basal B cell lines showed the highest fraction (33.3%). Interestingly, in a recent study it was proposed that breast tumors belonging to the HER2-enriched subtype often show the APOBEC mutational pattern [43]. However, even if our data set was limited and derived from cancer cell lines, additional studies on distinct APOBEC patterns in larger patient cohorts should be considered.

**Fig 5:**
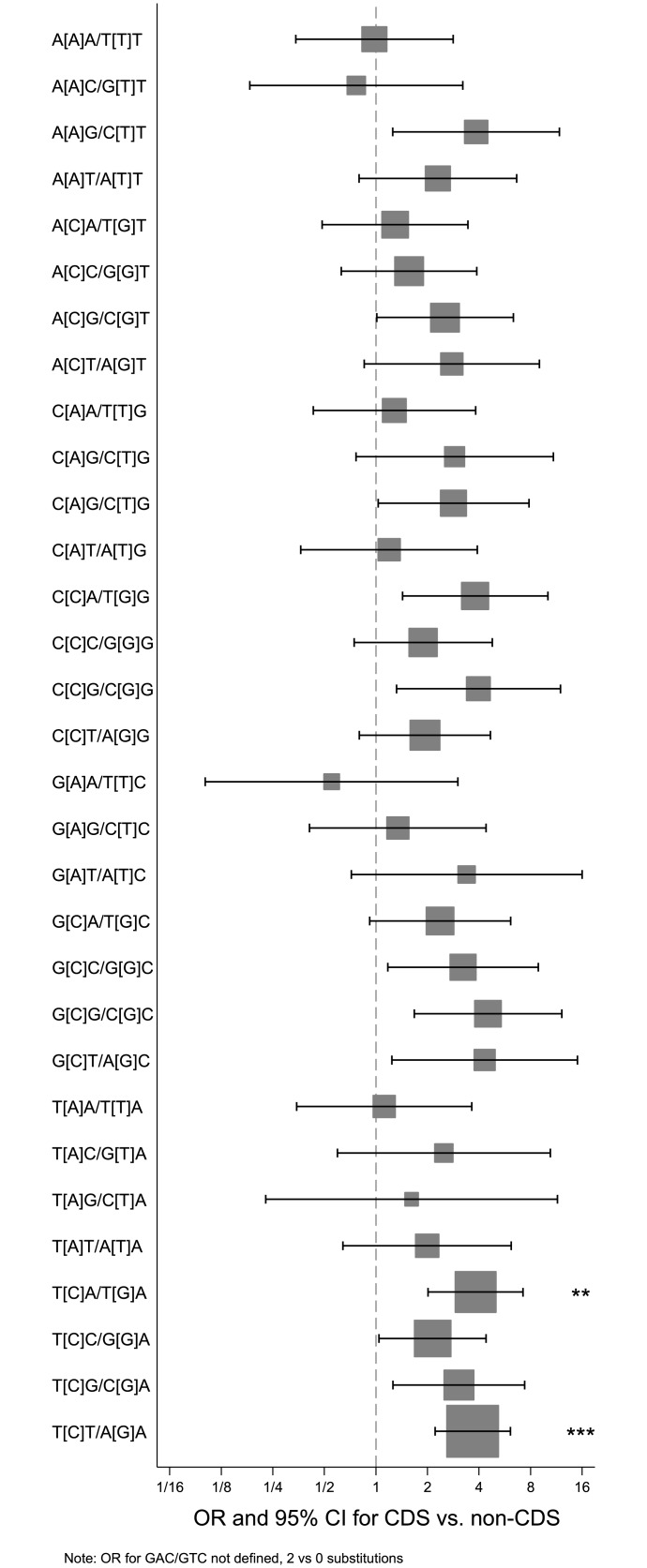
Genomic context of mutations in coding regions versus non-coding regions. Forest plot indicating the odds ratio (marker) and 95% confidence intervals (whiskers) for the trinucleotide context for base replacements (the center base within square brackets, both strands indicated) in coding regions (CDS) versus non-coding (non-CDS) regions. The dimensions of the squares are inversely proportional to the standard error (SE) of ln(odds ratio). ** P = 0.001; *** P<0.0001 (Bonferroni adjusted). Note that the odds ratio for G[A]C/G[T]C is undefined (2 substitutions in coding regions versus 0 in non-coding regions).

The pattern of adjacent bases, including the base before and after each SNV, was also investigated. Within the coding regions, SNVs occurred most frequently within the context of T[C]G/C[G]A and second most commonly at A[C]G/C[G]T, and in the non-coding regions these combinations were also the two most common, although in reverse order. Moreover, enrichment for these combinations were mainly seen in the Signatures 1B and 2 of the normalized breast cancer signatures [42]. The SNVs within the context of T[C]A/T[G]A and T[C]T/A[G]A were more frequent in the coding than in the non-coding regions (OR 3.8, 95% CI 2.0–7.2, P = 0.001; OR 3.7, 95% CI 2.2–6.1, P<0.0001, respectively) (Fig 5, S9 Table), and again, an enrichment for these combinations was seen particularly in the normalized Signatures 1B and 2. The number of each trinucleotide combination in the different regions was taken into account in the calculations. The most common trinucleotide combination, in absolute terms, was T[C]T/A[G]A in both coding and non-coding regions (n = 62 and n = 26, respectively).

## Conclusion

In conclusion, we have successfully used targeted sequencing of six basal-like breast cancer cell lines to identify 658 high confidence somatic mutations of which 315 are novel. Sanger sequencing confirmed 125 novel candidates, with a validation rate of 94% of the novel high confidence variants indicating that the majority of these are true. DNA copy number was estimated across the genome and agreed well to data from an orthologous method performed elsewhere. Although a limited number of cell lines were analyzed, mutational base replacement patterns may reflect complex mutational processes present in these model cell lines and deserves further study. Sanger sequencing showed good concordance to the exome data and highlighted the usefulness of reasonable variant call filtering thresholds and thresholds for calling a variant as somatic, as well as limitations in indel calling. Together, our results across 1,237 cancer-associated genes and the sequencing data will aid in the design and interpretation of biological experiments using these six commonly utilized basal-like breast cancer cell lines.

## Materials and Methods

### Cell lines and DNA

Original cell line stocks for HCC38, HCC1143, HCC1187, HCC1954, HCC1937 and HCC1395 were obtained directly from the American Type Culture Collection (ATCC), cultured according to Neve et al [17], and passaged minimally prior to harvesting for the present study. For each respective cell line, normal matched DNA derived from B lymphoblastoid cell lines (HCC38 BL, HCC1143 BL, HCC1187 BL, HCC1954 BL, HCC1937 BL, HCC1395 BL) were purchased from ATCC. Breast cancer **c**ells were harvested at approximately 75% confluency and genomic DNA was isolated using DNeasy Blood and Tissue Kit (Qiagen) according to the standard manufacturer protocol. The concentration of DNA was measured with the ND-1000 NanoDrop spectrophotometer (NanoDrop Technologies).

### Custom DNA SureSelect library

A custom SureSelect DNA library (Agilent Technologies) was constructed by selection of 1,237 genes suggested to be of importance primarily in breast cancer. All genes previously found mutated in breast cancer in the COSMIC (ftp://ftp.sanger.ac.uk/pub/CGP/cosmic/data_export/; date of access 2010/01/26) were selected and the list was expanded to include additional genes of interest as well as genes selected from the literature [22, 44–47]. All coding exons of selected genes were included in addition to 300 bp upsteam of the RefSeq transcript, the complete 5’UTR and the first 1200 bp of 3’UTR (human genome build hg18). All selected regions (20,674 separate target regions totaling 5,352,371 bp) were submitted to Agilent eArray (https://earray.chem.agilent.com/earray/) for probe design. The probes were 120 bp in length, designed end-to-end and with 1x coverage (on average 30 bp was targeted into each intron). Overall, 54,636 SureSelect baits were included, covering 6,556,320 bp, which corresponds to 0.22% of the human genome.

### Library preparation for sequencing

Target enrichment was performed on genomic DNA of all breast cancer cell lines and matched normal DNA. For each cell line, 3.6 μg of genomic DNA was sheared using the S220 Focused-Ultrasonicator Instrument (Covaris) with the following settings: Duty cycle 10%, Intensity 5, Cycles per burst 200, Time 3×2 min at 5°C. After purification, the fragment sizes were determined using the 2100 Bioanalyzer (Agilent Technologies) and on average fragments of 180 bp were obtained. Subsequently, NEBNext DNA Library Prep Master Mix Set I (New England Biolabs) was used for end-repair, dA-tailing and ligation of Genomic adaptors (Illumina) or custom tagged adaptors. Size selection was done using Agencourt AMPure beads (Beckman Coulter) to keep only fragments between 200–350 bp and this was verified by using the 2100 Bioanalyzer. Pre-capture PCR was performed in a total volume of 100 μl (divided in two tubes) with Phusion Master Mix 1x (Thermo Scientific), 250 nM of PE 1.0 or PE 2.0 primers together with half volume of the size selected DNA. PCR cycling conditions were as follows: Initial denaturation at 98°C for 2 min followed by 8 cycles of 98°C for 20 s, 65°C for 30 s and 72°C for 30 s and a final elongation at 72°C for 5 min. Capturing of selected genomic regions was performed according to SureSelect Hybridization protocol using the Agilent SureSelect Human All Exons protocol (Agilent Technologies). Briefly, biotinylated RNA library baits were hybridized in SureSelect hybridization buffer system and DNA regions of interest were captured by Dynabeads streptavidin-coated magnetic beads (Invitrogen). Post-hybridization enrichment was performed using 1x Herculase II reaction buffer, 1.25 mM each dNTPs, SureSelect GA PCR primers and Herculase II Fusion DNA polymerase with the following PCR conditions: 98°C for 2 min followed by 15 cycles of 98°C for 20 s, 57°C for 30 s and 72°C for 30 s and a final elongation at 72°C for 5 min. The samples were pooled and purified with Agencourt AMPure beads and the size of each library was validated on the 2100 Bioanalyzer (average length 275 bp) and the concentration was measured on Qubit (Invitrogen).

### Cluster generation and sequencing

Clusters were generated on a cBot instrument (Illumina). Single read sequencing of 75–80 bp was performed on a Genome Analyzer IIx instrument according to manufacturer's instructions. Raw TIF images were analyzed by Illumina Off-Line BaseCaller v1.6 and the base calling analysis was done using Bustard v1.6 (Illumina).

### Alignment and variant calling

Single reads were aligned to the human reference genome hg19 using bwa v0.5.9 [19], and Picard Tools v1.46 (http://picard.sourceforge.net/) were used to merge the tumor and matched normal BAM-files and to flag duplicates to ignore them in subsequent analysis. In the variant calling procedure, the analyzed regions were extended by 30 bp up- and downstream in the human genome, juxtaposed to the regions targeted by the SureSelect probes. Realignment (IndelRealigner) of targeted regions and quality score calibration (TableRecalibration) was done before variant calling of SNPs and indels using UnifiedGenotyper, all tools from the GenomeAnalysisTK v1.1–32 [20]. A minimum variant call quality score of 10 was required to call a variant. Known SNPs (dbSNP134) and indels (from the 1000 Genomes project) were used as input in IndelRealigner and known SNPs were used in TableRecalibration. GATK v3 best practices recommendations were followed with the following hard filters applied for SNPs: variant call quality < 40 || quality-by-depth (QD) < 5.0 || Homopolymer Run (HRun) > 5 || Fisher Strand bias (FS) > 200.0, and for indels: variant call quality < 40 || QD < 2.0 || Rank Sum Test for relative position in read of wild type vs alternative allele (ReadPosRankSum) < -20.0 || FS >200 (21). Variants were called as somatic if the most likely genotype determined by UnifiedGenotyper included the alternate allele for the tumor while the normal was determined as homozygous for the reference allele. Somatic variants that passed the variant call filters (see above), where the normal had an informative read depth ≥ 10 and ≤ 1 read supporting the alternative allele and the tumor had ≥ 3 reads supporting the alternate allele were considered “high confidence” somatic variants. All other somatic variants were considered “low confidence”.

### Sequencing statistics

The sequencing depth statistics was calculated using GATK DepthofCoverage v2.8–1 [20], in the targeted regions of interest covered by our custom SureSelect bait library. Picard Tools v1.46 (http://picard.sourceforge.net/) were used to derive the read metrics per sample.

### Annotation of somatic mutation and copy number variants

All somatic variants were annotated in Annovar [48] with functional-, regional-, and filter-based annotations from selected databases: RefSeq gene annotations, dbSNP138, allele frequencies in NCI60 [49], presence in the TCGA BRCA study (mutation calling file level2.5.1.0) [14], MutationTaster and Mutation Assessor scores (LJB2.3, dbNSFP) [38, 39, 50], CADD (Combined Annotation Dependent Depletion) c-scores [40], PhyloP scores (phastConsElements46way), and ClinVar (20140211) [51]. All cell lines were annotated by previously known somatic mutations entered in the COSMIC database v68 [21]. The combined list of genes affected by mutational and copy number aberrations were annotated with the KEGG pathway categorization using the DAVID Bioinformatics Resources 6.7 tool with the whole genome list for *Homo sapiens* as background [33, 52].

### Sanger sequencing validation of somatic variants

In total, 172 novel mutations were selected for validation by Sanger sequencing. Both high and low confidence mutations were selected, however, a QD score >3 was required for the low confidence variants to be included. Moreover, 15 variants found both in COSMIC and by our pipeline and 36 variants annotated as mutated in COSMIC but not found in our data were also selected for Sanger sequencing validation. Whenever possible, previously published primers were used for PCR amplification [53], otherwise, primer pairs were designed. Primer design was performed in Primer3 v0.4.0 with Santa Lucia 1998 settings GC clamp set to 1 [54]. The primers were designed not to be within 30 bp of the variant and the primer closest to the variant was tagged with M13 for sequencing. All primers were synthesized by Integrated DNA Technologies (IDT). PCR reactions were performed in a total volume of 10 μL with Phusion Master Mix 1x (Thermo Scientific), 200 nM of each primer, 2% of DMSO and 10 ng of template DNA and the PCR was run with the following cycling conditions: initial denaturation at 98°C for 2 minutes followed by 11 cycles of 98°C for 10 s, 70°C (-1°C/cycle) for 30 s and 72°C for 15 s and then 29 cycles of 98°C for 10 s, 60°C for 30 s and 72°C for 30 s and a final elongation at 72°C for 5 min. Both tumor and matched normal DNA were used as input for each primer pair assay in separate reactions. All PCR products were purified and sequenced by Beckman Coulter Genomics and Sequencher v5.0.1 (Gene Codes Corporation) was used to evaluate the chromatograms of the tumor and normal sample traces in parallel.

### Copy number variation analysis

Copy number variation (CNV) was estimated using the tool CONTRA v2.0.4, a software specifically designed for CNV detection in targeted resequencing data [31]. Deduplicated and realigned bam-files for each tumor and the matched normal sample were used as input data. CONTRA was run with default settings except for the maxRegionSize 150 and targetRegionSize 100. The adjusted mean log ratios derived from CONTRA derived from CONTRA were filtered on coverage (regions with coverage <20 in the matched normal sample were removed) and segmented using the Bioconductor software GLAD [32]. In the GLAD algorithm the bandwidth = 1, qlambda = 0.999999, lambdabreak = 8, lambdacluster = 8, lambdaclusterGen = 40 and alpha = 1e-3 were applied. Copy numbers estimations (log2 ratios) for individual genes were calculated for genes larger than 400bp. To score homozyogous deletions and high level gains that were present in our data, log2 thresholds of <-2 and >2, respectively, were applied. Only genes with all exons passing the threshold were included as high level gains, and pass-threshold deletions of regions ≥ 400bp were considered as homozygous deletions only if exonic regions were included. To make a comparison of the results from CONTRA to publicly available CNV data, segmented Affymetrix 6.0 SNP array data was downloaded from the Cancer Cell Line Encyclopedia [16]. The two methods for copy number estimation were compared by correlating each of the CONTRA copy number values per segmented window to the corresponding copy number values derived from the Affymetrix SNP array data. The mean genomic position per window in the segmented CONTRA data were used to lookup the corresponding copy number value in the Affymetrix data. Pearson correlation was used to compare the data from the two methods.

### Base replacements and trinucleotide combinations

The base replacement frequencies were calculated by using the number of G/C or T/A bases as denominator and the number of respective base replacements as numerator, in coding versus non-coding regions. The trinucleotide frequencies were calculated by using the number of each trinucleotide combination as denominator and the number of SNVs within each context as numerator, in coding versus non-coding regions. Only SNPs passing the variant call filters were included in the analysis (see above). The statistical calculations were performed by using logistic regression interaction modeling (between samples) and all p-values were corrected with Bonferroni adjustment.

### Analysis tools

The software BedTools v2.17.0, and Samtools v0.1.19 together with custom scripts in R v3.1.0, Stata/SE v13.1 (StataCorp LP) and Python v2.7 were used to implement the analysis, for statistical calculations, and for plotting of the data.

### Data availability

The sequencing data BAM files and target region BED file are publically available from the Dryad repository: http://doi.org/10.5061/dryad.cg40g.

## Supporting Information

S1 Fig Copy number plots of targeted sequencing data. (a-f) Whole genome plots of CONTRA copy number (log2 ratios) derived from targeted sequencing data for respective cell line in comparison to segmented Affymetrix 6.0 copy number data. For the sequencing data, the black datapoints are the CONTRA adjusted mean log2 ratios, and red datapoints are the CONTRA/GLAD segmented copy log2 ratios. For the Affymetrix 6.0 data, blue datapoints are the segmented copy number data.
(TIF)

S2 Fig Correlation plots for targeted sequencing CNVs versus Affymetrix 6.0 for respective cancer cell line. Segmented CONTRA copy number data derived from targeted sequencing data versus segmented CCLE Affymetrix 6.0 copy number data are shown in the plots (Pearson correlation 0.82 to 0.96 for all compared cell lines; P<0.0001).
(TIF)

S1 Table Genes included in targeted capture regions. (XLSX)

S2 Table Sequencing statistics. (XLSX)

S3 Table All somatic variants detected by GATK UnifiedGenotyper and annotated by Annovar. (XLSX)

S4 Table Sanger sequencing validation of novel mutations, primer details. (XLSX)

S5 Table Sanger sequencing validation of mutations both detected in our targeted sequencing analysis and in COSMIC v68. (XLSX)

S6 Table Sanger sequencing validation of mutations in COSMIC v68 but not detected by in our targeted sequencing analysis. (XLSX)

S7 Table Estimated average copy number for individual genes. (XLSX)

S8 Table Number of mutations in coding respective non-coding regions for respective base replacement. (XLSX)

S9 Table Number of mutations within respective genomic context in coding and non-coding regions. (XLSX)
