# Supplementary figures and images for: Mutation Screening of 1,237 Cancer Genes across Six Model Cell Lines of Basal-Like Breast Cancer
Source: PLoS One. 2015 Dec 15;10(12):e0144528. doi: 10.1371/journal.pone.0144528 (PMC4684399; doi:10.1371/journal.pone.0144528)

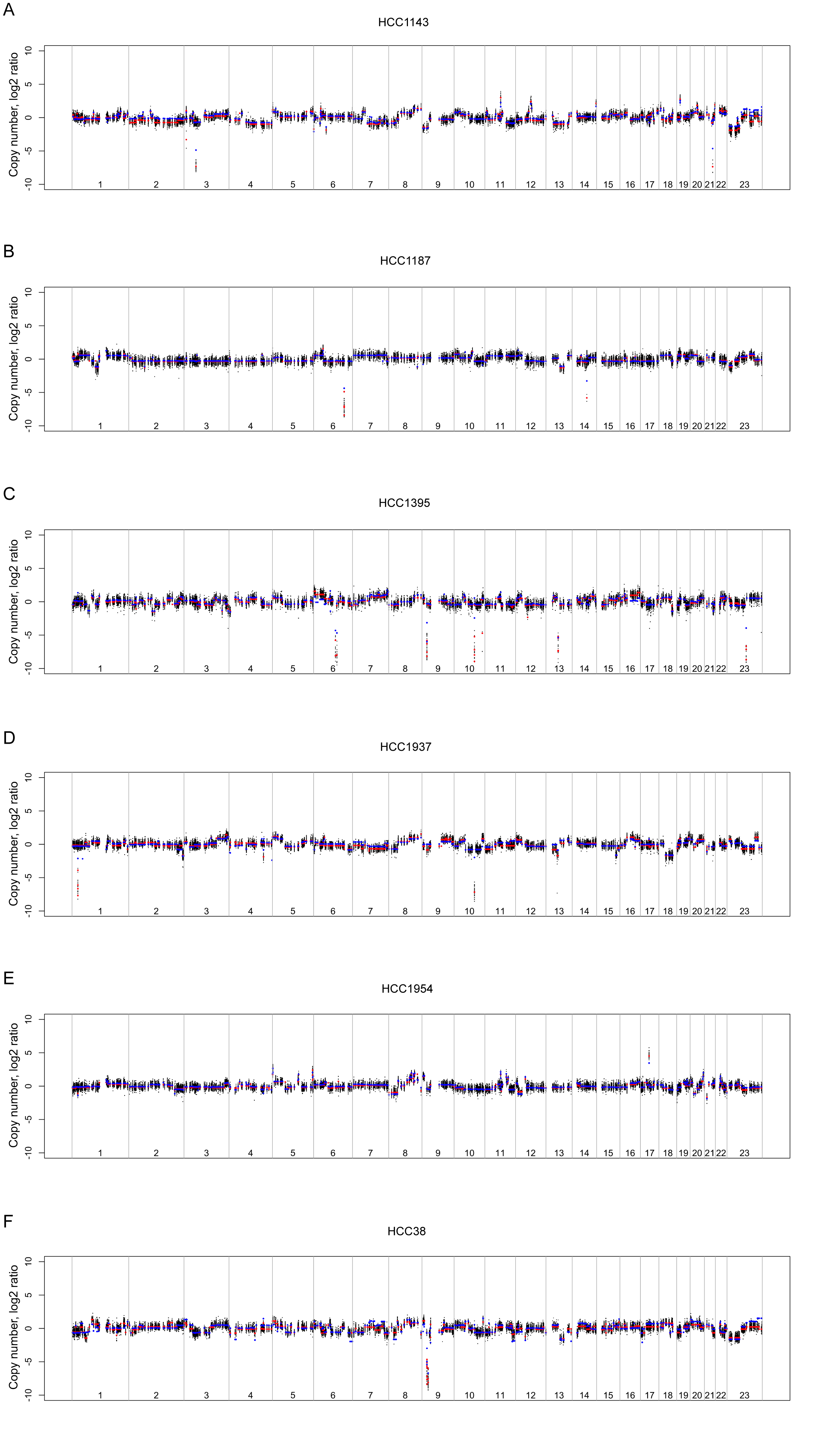

Supplement: S1 Fig — (a-f) Whole genome plots of CONTRA copy number (log2 ratios) derived from targeted sequencing data for respective cell line in comparison to segmented Affymetrix 6.0 copy number data. For the sequencing data, the black datapoints are the CONTRA adjusted mean log2 ratios, and red datapoints are the CONTRA/GLAD segmented copy log2 ratios. For the Affymetrix 6.0 data, blue datapoints are the segmented copy number data. (TIF) [file pone.0144528.s001.tif]

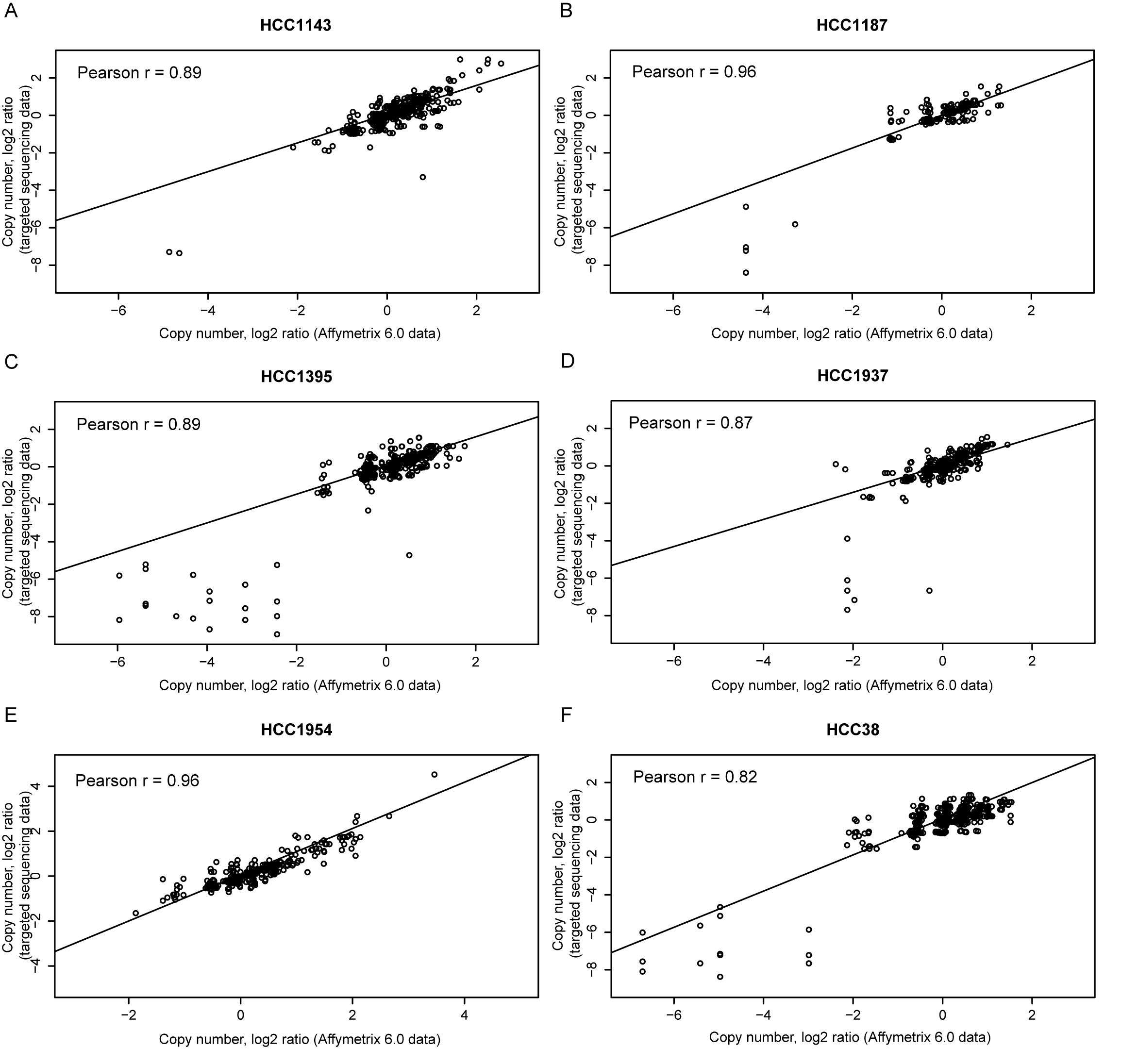

Supplement: S2 Fig — Segmented CONTRA copy number data derived from targeted sequencing data versus segmented CCLE Affymetrix 6.0 copy number data are shown in the plots (Pearson correlation 0.82 to 0.96 for all compared cell lines; P<0.0001). (TIF) [file pone.0144528.s002.tif]
